# Luteal and placental function in the bitch: spatio-temporal changes in prolactin receptor (PRLr) expression at dioestrus, pregnancy and normal and induced parturition

**DOI:** 10.1186/1477-7827-9-109

**Published:** 2011-08-03

**Authors:** Mariusz P Kowalewski, Erika Michel, Aykut Gram, Alois Boos, Franco Guscetti, Bernd Hoffmann, Selim Aslan, Iris Reichler

**Affiliations:** 1 Institute of Veterinary Anatomy, Vetsuisse Faculty, University of Zurich, Zurich, Switzerland; 2 Section of Small Animal Reproduction, Clinic of Reproductive Medicine, Vetsuisse Faculty, University of Zurich, Zurich, Switzerland; 3 Institute of Veterinary Pathology, Vetsuisse Faculty, University of Zurich, Zurich, Switzerland; 4 Clinic for Obstetrics, Gynecology and Andrology of Large- and Small Animals, Justus-Liebig University, Giessen, Germany; 5 Clinic for Obstetrics and Gynecology, Faculty of Veterinary Medicine, University of Ankara, Ankara, Turkey

## Abstract

**Background:**

Endocrine mechanisms governing canine reproductive function remain still obscure. Progesterone (P4) of luteal origin is required for maintenance of pregnancy. Corpora lutea (CL) are gonadotrop-independent during the first third of dioestrus; afterwards prolactin (PRL) is the primary luteotropic factor. Interestingly, the increasing PRL levels are accompanied by decreasing P4 concentrations, thus luteal regression/luteolysis occurs in spite of an increased availability of gonadotropic support. PRL acts through its receptor (PRLr), the expression of which has not yet been thoroughly investigated at the molecular and cellular level in the dog.

**Methods:**

The expression of PRLr was assessed in CL of non-pregnant dogs during the course of dioestrus (days 5, 15, 25, 35, 45, 65 post ovulation; p.o.) as well as in CL, the utero/placental compartments (Ut/Pl) and interplacental free polar zones (interplacental sites) from pregnant dogs during the pre-implantation, post-implantation and mid-gestation period of pregnancy and during the normal and antigestagen-induced luteolysis. Expression of PRLr was tested by Real Time PCR, immunohistochemistry and *in situ *hybridization.

**Results:**

In non-pregnant CL the PRLr expression was significantly upregulated at day 15 p.o. and decreased significantly afterwards, towards the end of dioestrus. CL of pregnancy showed elevated PRLr expression until mid gestation while prepartal downregulation was observed. Interestingly, placental but not interplacental expression of PRLr was strongly time-related; a significant upregulation was observed towards mid-gestation. Within the CL PRLr was localized to the luteal cells; in the Ut/Pl it was localized to the fetal trophoblast and epithelial cells of glandular chambers. Moreover, in mid-pregnant animals treated with an antigestagen, both the luteal and placental, but not the uterine PRLr were significantly downregulated.

**Conclusions:**

The data presented suggest that the luteal provision of P4 in both pregnant and non-pregnant dogs may be regulated at the PRLr level. Furthermore, a role of PRL not only in maintaining the canine CL function but also in regulating the placental function is strongly suggested. A possible functional interrelationship between luteal P4 and placental and luteal PRLr expression also with respect to the prepartal luteolysis is implied.

## Background

Regulation of reproductive function in the domestic dog (*Canis familiaris*) is governed by many, as yet not fully understood, species-specific mechanisms. Most of the knowledge available so far relates to clinical observations. Thus, the production of progesterone of follicular origin is observed already prior to ovulation at the end of proestrus and reaches the levels of about 5 ng/ml in peripheral blood plasma at the time of ovulation [1]. The Corpus luteum (CL) phase and, associated with it, luteal progesterone secretion is virtually identical in pregnant and cyclic animals until shortly before parturition [1,2]. Hence, during the formation of the CL (until day 15-20 post ovulation) a strong rise in the concentration of peripheral progesterone levels takes place. Afterwards, progesterone levels start to decrease gradually until approximately day 60 of the luteal life-span when a steep prepartal progesterone decline is observed in pregnant animals (prepartal luteolysis) as a prerequisite for parturition [3]. In non-pregnant dogs in contrast, there is a progressive decrease of progesterone levels during the extended luteal regression that can last for up to 1-3 months until the peripheral P4 will eventually reach levels < 1 ng/ml and the cycle enters anoestrus [1,2,4,5]. Interestingly and in contrast to polyestrus species (e.g. cattle, pig), where ovarian cyclicity depends upon the periodic production of the endometrial luteolysin (PGF2α), at least in non-pregnant bitches, the uterus does not regulate luteal function since normal ovarian function is observed following hysterectomy [6,7]. In pregnant dogs, however, the prepartal progesterone decline coincides with an increase in PGF2α levels in maternal blood [3]. This seems to originate from the dramatically increased expression of cyclooxygenase 2 (COX2) in the fetal trophoblast [8] and strongly implies a functional role of PGF2α in relation to the onset of parturition [3]. The withdrawal of progesterone and alterations in the feto-maternal communication at the level of maternal decidual- and fetal trophoblast cells seem to play a signalling role in this process [8]. The observation that antigestagen-mediated blocking of progesterone receptors results in the activation of an endocrine cascade within the luteal and placental prostaglandin system similar to that observed during normal prepartal luteolysis further supports this hypothesis [8,9].

Mechanisms regulating corpus luteum function, both in pregnant and non-pregnant bitches, have been widely discussed. During the first 2-6 weeks of dioestrus the gonadotropic support is not required for luteal maintenance [10-12]. Recently, a possible luteotropic role for prostaglandin E2 (PGE2) produced locally, within the corpus luteum (CL) during this period of time has been suggested [13]. Thereafter, in the second half of pregnancy, hypophysectomy, and hence deprivation of gonadotropic support leads to cessation of luteal function [10-12]. Serum concentrations of PRL remain mostly low during the first half of pregnancy, at approximately 5-6 weeks of gestation they begin to increase constantly towards parturition [14]. In non-pregnant animals serum concentrations of PRL remain low during most of the course of dioestrus and increase 2 to 3 fold around day 50 post ovulation, which is the time when parturition would have occurred [7,14,15]. Similarly, an increased availability of the luteinizing hormone (LH) has been observed in bitches when P4 concentrations were declining during the course of dioestrus [6,16]. Therefore, the luteal regression and/or luteolysis occurs in spite of an increased availability of gonadotropic support.

In contrast to species in which provision of LH seems to be essential for luteal function, e.g. cattle [17], pigs [18] and horses [19], in the dog PRL appears to be the main gonadotropic factor involved in maintenance of the CL function, and hence pregnancy from the second third of dioestrus onwards [11,20,21]. While sensitivity of the canine ovary to prolactin withdrawal can occur as early as at day 25 after the preovulatory LH peak and can lead to permanent luteal regression [22], blocking of LH function results only in a transient progesterone decrease, both in pregnant and non-pregnant animals [11,23]. LH does not seem to stimulate progesterone secretion directly but, especially during the late luteal phase, it is capable of increasing prolactin concentrations [23]. From this observation a possible indirect role of LH in regulating canine luteal function acting as a mediator in PRL secretion has been suggested [23]. On the other side neither a direct stimulatory effect of PRL on plasma progesterone secretion nor a noticeable effect on plasma LH levels was seen [21]. Thus, although PRL appears to be an essential luteotropin from mid-luteal phase in both pregnant and non-pregnant dogs, its role seems to be directed more towards slowing down luteal regression than stimulating progesterone production [21].

PRL acts through the PRL receptor (PRLr), a single membrane-bound protein of the class 1 of the cytokine/PRL/GH receptor family [24,25]. Several isoforms of PRLr have been identified in different tissues of mammals (see reviewed by [26]). For example in the rat there are three major PRLr isoforms; the short, intermediate and long one have been described [26]. In mice three short and one long isoform [27], in cattle one short and one long [28,29], and in humans five isoforms are known [30]. The different PRLr isoforms can result from utilizing different PRLr promoters for initializing of transcription as well as can be expressed as alternative splice variants [31,32]. The different length and structure, especially concerning the intracellular domain of the PRLr isoforms, as well as the ratio of short to long isoforms, result in altered signalling properties and determine the tissues sensitivity to PRL [33,34]. Among the various signalling cascades that are activated through this receptors family, activation of tyrosine kinases of the Janus kinase (JAK) family and subsequent phosphorylation/activation of the cytoplasmic signal transducers and activators of transcription (STATs) is considered the major signalling pathway [26,33].

The presence of that many isoforms of the PRLr indicates multiple possibilities for their involvement in regulating endo-, para- and/or autocrine functions of targeted organs. They are probably also characterized by a high species dependent diversity.

As for the dog, and concerning the presence of PRLr in canine reproductive tissues, Fernandes et al. [15] reported about the concentrations of the binding sites for prolactin in the luteal membranes isolated from tissue homogenates obtained from non-pregnant dogs during the course of dioestrus. They tended to be maximal during the first 40 days of the luteal life-span and were generally lower thereafter, when serum and luteal concentrations of P4 were decreasing [15]. Concomitantly with the decrease of peripheral P4 levels a decrease in expression of the steroidogenic acute regulatory protein (StAR) occurs [9,35]. This suggests that production of the luteal P4 in dog might be controlled by the substrate provision at the level of cholesterol transfer from the outer to the inner mitochondrial membrane. The role of the upstream operating regulatory factors, however, remains open and needs to be elucidated. Since, acting through its receptor, PRL is a vital luteotropic hormone from the mid-luteal phase in the dog, PRLr obviously becomes one of the possible candidates. To our knowledge its expression in canine reproductive tissues has not yet been investigated at the molecular and cellular level. Therefore, the current study was undertaken to obtain first information on the expression and localization of the PRLr in canine CL from pregnant and non-pregnant dogs. Even if devoid of steroidogenic capacity [1], canine placenta appears to be an endocrine organ actively responding to the circulating progesterone levels [8] and showing thereby an immediate functional interrelationship with CL-derived steroids. Hence, in order to test if canine placenta could be a possible target for PRL we have also investigated the spatio-temporal expression of PRLr in the utero-placental compartments (Ut/Pl).

## Methods

### Tissue collection and preservation

Clinically healthy and sexually mature bitches of various breeds (aged 2-8 years) were used for this study. All experiments were in accordance with the respective animal welfare legislation ((permit no. II 25.3-19c20-15c GI 18/14 and VIG3-19c-20/15c GI 18,14 (Justus-Liebig University, Giessen) and permit no. Ankara 2006/06 (Faculty of Veterinary Medicine, University of Ankara)).

#### Non-pregnant bitches

Groups of five bitches each were ovariohysterectomized on days 5, 15, 25, 35, 45 and 65 after ovulation (p. o.). Ovarian function was monitored by determining P4 in 2-3 day intervals. The day of ovulation was defined as the day when plasma progesterone concentration reached 5 ng/mL [1].

#### Pregnant bitches

Animals were divided into four groups and subjected to ovariohysterectomy at following days of pregnancy: 8-12 (pre-implantation; n = 5), 18-25 (post-implantation; n = 5), 35-45 (mid-gestation; n = 5) and prepartal luteolysis; n = 3.

For all bitches the day of mating (day 0) was recorded. In the pre-implantation group pregnancy was confirmed by detecting embryos in uterine flushing. For the prepartal luteolysis - group, P4 was determined every 6 h from day 58 of pregnancy on; at P4 levels continuing to decrease below 3 ng/ml within two consecutive measurements ovariohysterectomy was performed.

Additionally, mid-pregnant dogs (days 40-45 of pregnancy, n = 10) were treated with the antiprogestin Aglepristone^® ^[10 mg/Kg bw (2×/24 hrs apart)]. In this group ovariohysterectomy was performed 24 hrs and 72 hrs after the 2^nd ^treatment.

Immediately after ovariohysterectomy Corpora lutea (CL) from pregnant and non-pregnant animals as well as the utero/placental compartments (Ut/Pl) and samples from the interplacental free polar zones of animals sampled at the post-implantation and mid-gestation periods were trimmed off the surrounding connective tissues (there was no tissue material available for the experiments from the interplacental sites from dogs during prepartal luteolysis). The tissue samples were then incubated for 24 h in RNAlater^® ^(Ambion Biotechnologie GmBH, Wiesbaden), an aqueous reagent for stabilization of cellular RNA (ratio mass:volume = 1:6) and subsequently stored at -80°C until total RNA isolation. For immunohistochemistry (IHC) and In situ hybridization (ISH) samples were fixed in 10% neutral phosphate buffered formalin for 24 h, frequently washed with PBS during one week, dehydrated in a graded ethanol series and embedded in paraffin-equivalent Histo-Comp (Vogel; Giessen, Germany).

As already published in [36] and kindly provided by Dr. Paula Papa of the Sector of Anatomy of the Faculty of Veterinary Medicine of the University of Sao Paulo the corresponding peripheral progesterone levels in non-pregnant animals were: 41.3 ± 11.7 ng/ml at day 5 p.o., 53.7 ± 2.6 ng/ml at day 15 p.o., 42.0 ± 20.6 ng/ml at day 25 p.o., 30.1 ± 4.31 ng/ml at day 35 p.o. and 22.9 ± 5.9 at day 45 p.o.. Here we extended the data including day 65 p.o. with expected low progesterone levels (6.4 ± 1.59 ng/ml). For the pregnant animals following corresponding mean progesterone concentrations were shown in [9]; 35.71 ± 7.9 ng/ml in the pre-implantation period, 29.73 ± 13.23 ng/ml in the post-implantation period, 13.32 ± 8.66 ng/ml at mid-gestation and 2.07 ± 0.99 ng/ml during the prepartal progesterone decline. After Alizine^® ^application and as presented in [9]: before the first treatment 15.11 ± 6.7 ng/ml; at the second Aglepristone treatment 13.61 ± 8.2 ng/ml; 5.1 ± 2.7 ng/ml 24 h later and 2.33 ± 1.44 ng/ml, respectively 1.2 ± 0.6 ng/ml 48 and 72 h later.

### RNA isolation and homology cloning of canine-specific partial prolactin receptor (PRLr) sequence

Trizol^®^-Reagent (Invitrogen, Carlsbad, CA) was used to isolate total RNA from CL and Ut/Pl compartments. Subsequently, DNase-treatment with RQ1 RNase-free DNase (Promega, Dübendorf, CH) was performed to eliminate genomic DNA contaminations. Both procedures were according to the manufacturers' instructions. Reverse transcription reagents were purchased from Applied Biosystems, Foster City, CA, USA; random hexamers were used as primers for the cDNA synthesis. Of each sample 100 ng of DNase-treated total RNA were used. Reactions were carried out in an Eppendorf Mastercycler Thermal Cycler (Vaudaux-Eppendorf AG, Basel, CH) using our previously published protocol [37].

**Figure 1:**
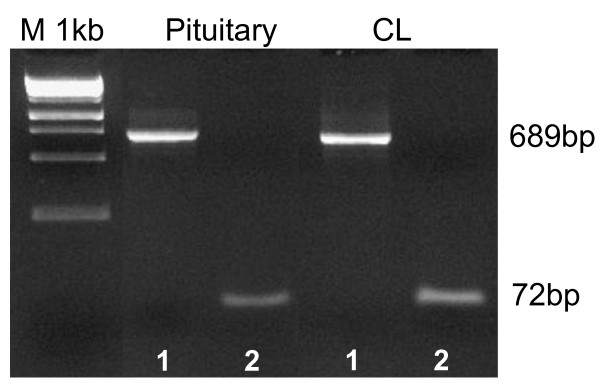
**Expression of canine PRLr (1) and GAPDH (2) in anterior pituitary (positive control) and corpus luteum by conventional RT-PCR**. The amplified PRLr fragments were used for the cloning procedure.

To date the canine-specific PRLr sequence has not yet been characterized. Thus, alignment of the known PRLr homologues with an online available genomic sequence was performed in order to derive the canine-specific primers for PRLr. Using the primer pair: forward 5'-GTG GGA GAC TCA TTT TGC TG-3' and reverse 5'-CGA AGT AGG GGA TTT TGC C-3', a 689 bp PCR fragment of partial canine PRLr was succesfully amplified (Figure 1). The annealing temperature was 56°C. The hot start PCR reaction with the GeneAmp Gold RNA PCR Kit (Applied Biosystem, Foster City, CA, USA) was according to the previously published protocol [37]. Primers were ordered from Microsynth AG, Balgach, CH. After separation on a ethidium bromide stained 2% agarose gel, PCR products were purified using the Qiaex II gel extraction system (Qiagen GmbH Hilden, Germany), ligated into a pGEM-T vector (Promega, Dübendorf, CH), transformed and amplified in XL1 BLUE competent cells (Stratagene, la Jolla, CA, USA) and finally sequenced on both strands (Microsynth, Balgach, CH) with T7 and Sp6 sequencing primers. Autoclaved water instead of RNA and the so-called RT minus controls were used as negative controls. Integrity of RNA and the assay procedure were tested by amplification of the housekeeping gene *Gapdh *using following primers: forward: 5'-GCT GCC AAA TAT GAC GAC ATC A-3'and reverse: 5'-GTA GCC CAG GAT GCC TTT GAG-3'. For positive control a PCR reaction was performed using total RNA isolated from canine pituitary gland confirming the identity of the CL- and Ut/Pl- isolated sequence with the hypohysal PRLr. Finally, the cloned sequence was submitted to the GenBank with accession number: HQ267784: Canis lupus familiaris prolactin receptor mRNA, partial cds.

### Semi-quantitative RT-PCR and data evaluation

The semi-quantitative Real Time (Taq Man) PCR was performed using an automated fluorometer ABI PRISM^® ^7500 Sequence Detection System (Applied Biosystems, Foster City, CA) according to our previously described protocol [37]. DNase-treatment and cDNA synthesis as well as negative controls were as for qualitative PCR. Samples were run in duplicates with Fast Start Universal Probe Master (ROX) ^® ^from Roche Diagnostics. Three different independent endogenous references (GAPDH, 18SrRNA and cyclophilin A) were used for the semi-quantitation protocol. The primers and probes sequences were as follows: *Prlr *(forward): 5'-GGA TCT TTG TGG CCG TTC TTT-3', *Prlr *(reverse): 5'-AAG GAT GCA GGT CAC CAT GCT AT-3', *Prlr *(TaqMan Probe): 5'-ATT ATG GTC GTA GCA GTG GCT TTG AAA GGC-3' (gene bank accession number HQ267784); *Gapdh *(forward): 5'-GCT GCC AAA TAT GAC GAC ATC A-3', *Gapdh *(reverse): 5'-GTA GCC CAG GAT GCC TTT GAG-3', *Gapdh *(TaqMan Probe): 5'-TCC CTC CGA TGC CTG CTT CAC TAC CTT-3' (gene bank accession number AB028142); *18SrRNA *(forward): 5'-GTC GCT CGC TCC TCT CCT ACT-3', *18SrRNA *(reverse): 5'-GGC TGA CCG GGT TGG TTT-3', *18SrRNA *(TaqMan Probe): 5'-ACA TGC CGA CGG GCG CTG AC-3' (gene bank accession number FJ797658). The primers and the 6-carboxyfluorescein (6-FAM) and 6-carboxytetramethylrhodamine (TAMRA) labelled probes were from Microsynth, Balgach, CH. The commercially available canine specific cyclophilin A TaqMan Gene Expression Assay was ordered from Applied Biosystems, Foster City, CA, USA (Prod. No. Cf03986523-gH).

Measurement of the efficiency of the PCR reactions using the CT slope method and the relative quantification of PRLr expression using the comparative CT method (ΔΔCT) were according to previously described protocols [37,38]. In order to confirm the specificity of the PCR reaction, selected PCR products were commercially sequenced (Microsynths, Balgach, CH).

To test for an effect of the observational group on PRLr mRNA-levels, a parametric one-way analysis of variance (ANOVA) was applied. In case of p < 0.05 multiple comparison post-tests were performed. Those were: Tukey-Kramer multiple comparison test in experiments showing the expression of PRLr during the course of pregnancy and in luteal samples from non-pregnant dogs and Dunnett's multiple comparison test in experiments showing the PRLr expression after Aglepristone^®^-induced luteolysis. In the latter the results present the fold change in PRLr expression compared to its mRNA-levels at mid-gestation. Numerical data were presented as the mean ± standard deviation. For all tests statistical software program, GraphPad 3.06 (GraphPad Software, Inc., San Diego, California, USA) was used.

### Immunohistochemical detection of prolactin receptor (PRLr)

The standard immunoperoxidase detection method was as previously described [37,39]. The antiserum (dilution 1:50) was polyclonal goat antigen affinity-purified IgG fraction against human PRLr, R&D Systems Europe Ltd. Goat IgG irrelevant antibodies I-5000 from Vector Laboratories Inc., Burlingame, CA94010, CA, USA were used as the negative/isotype control. Unspecific binding sites were blocked with 10% horse serum in IHC/0.25% Triton X buffer. The secondary antibody was the biotinylated horse anti goat IgG BA-9500 from Vector Laboratories diluted 1:100. The peroxidase activity was detected with DAB substrate Kit according to the manufacturer's instruction (Dako North America, Inc.).

### In situ hybridization (ISH)

PCR products of 280 bp size generated with primers: forwards: 5'-GTG GGA GAC TCA TTT TGC TG-3' and reverse: 5'-AGG AAC TGG TGG AAG GAT G-3' were used as template for subsequent synthesis of cRNA probes. The PCR products were separated on an agarose gel, cut out, purified with the Qiaex II gel extraction system (Qiagen GmbH Hilden, Germany), and cloned into pGEM-T vector (Promega). After amplifying in XL1 BLUE competent cells (Stratagene, la Jolla, CA, USA) plasmids were linearized by single-digestion with restriction enzymes NcoI and Not (New England Biolabs, Frankfurt, Germany) for antisense- and sense-cRNA, respectively. The subsequent non-radioactive ISH was performed on paraffin-embedded sections of canine utero/placental section according to the previously published protocol [8,39].

## Results

### Expression of PRLr in Corpus luteum of pregnant and non-pregnant animals

Canine-specific sequence of PRLr has not been published prior to our work. Thus, cloning and sequencing of canine PRLr-cDNA were a prerequisite before investigating its expression pattern in canine reproductive tissues. The PRLr sequences isolated from luteal and/or utero/placental tissues were 100% identical with the sequence retrieved from pituitary.

**Figure 2:**
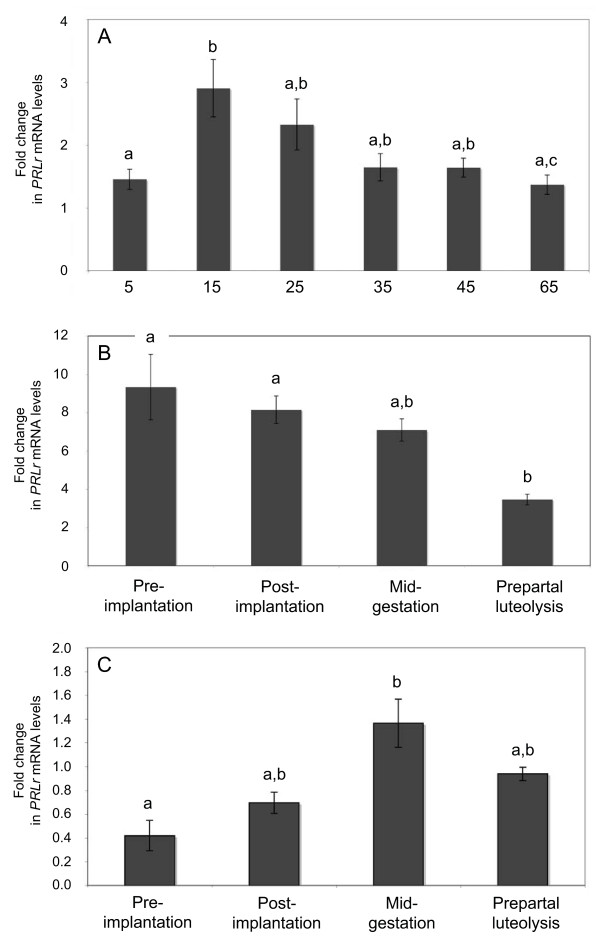
**Time-dependent expression of canine prolactin receptor (PRLr) as determined by Real Time (TaqMan) PCR (mean ± SD)**. (A) Cyclic CL, day 5-65 after ovulation. (B) CL of pregnancy. (C) Utero/placental compartment throughout pregnancy. Bars with different letters differ either at p < 0.05 (A,B) or at p < 0.01 (C).

Expression of PRLr mRNA and protein was detected in CL of pregnant and non-pregnant dogs at all time points examined. In non-pregnant dogs expression of mRNA followed a highly significant effect of time (p < 0.0103). The highest expression was at the beginning of the dioestrus, at day 15 p.o.. Afterwards, it decreased gradually and was significantly (p < 0.05) downregulated by 2.1-fold at day 65 p.o (Figure 2a).

There was also a significant effect of time for the expression of the luteal PRLr during pregnancy (p < 0.0140). The highest expression was observed during pre-implantation period (days 8-12) and post-implantation (days 18-25 of pregnancy) and decreased significantly by 2.4-fold (p < 0.05) during prepartal luteolysis (Figure 2b).

**Figure 3:**
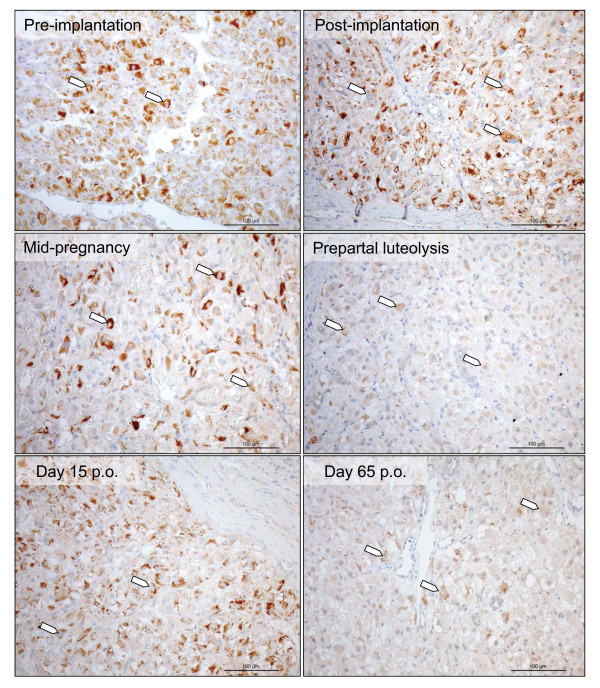
**Immunohistochemical localization of PRLr in canine CL during pregnancy from the pre-implantation period until prepartal luteolysis and on days 15 and 65 post-ovulation in non-pregnant animals; (open arrowheads) lutein cells**.

At the protein level as determined by immunohistochemistry, the expression of PRLr was localized mostly to the luteal cells. Signals were detected at all stages of the luteal life-span and the staining pattern reflected that of the mRNA-expression. Thus, the strongest signals were observed at the beginning and weaker towards the end of the luteal phase (Figure 3). The weak signals observed within the blood vessels throughout the luteal life-span can either be attributed to the lower expression and hence, detection limit of PRLr in the vessels or to the background staining.

### Expression of PRLr in canine utero/placental compartment and interplacental polar zones

**Figure 4:**
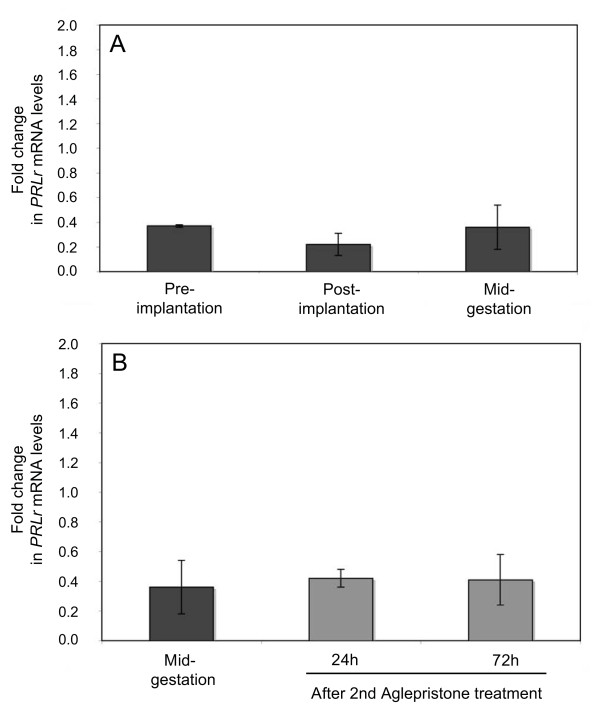
**Expression of PRLr as determined by Real Time (TaqMan) PCR (mean ± SD) in the interplacental polar zones (interplacental sites of uterus) from the pre-implantation period until mid-gestation (A) and during Aglepristone^®^- induced luteolysis (B; compared with the mid-gestation group as non-treated control)**.

The expression of PRLr within the utero/placental compartment was highly time-dependent. (p = 0.0077). The lowest expression was observed during the pre-implantation period. With the formation of the placenta levels of PRLr-mRNA started to increase and reached significantly higher level (p < 0.01) during the mid-gestation period. The decrease prior to parturition was not significant (p > 0.05) (Figure 2C). These observations led us to speculate on the source of this increased PRLr-mRNA expression within the utero/placental compartment coinciding with the intense placental development and prompted us to investigate the expression of PRLr-mRNA in the interplacental free polar zones (uterine compartment). However, as revealed by the Real Time PCR, no statistically significant differences (p > 0.05) were observed for the expression of the target gene in this compartment in samples derived from the post-implantation and mid-gestation period if compared with the pre-implantation period (Figure 4A).

**Figure 5:**
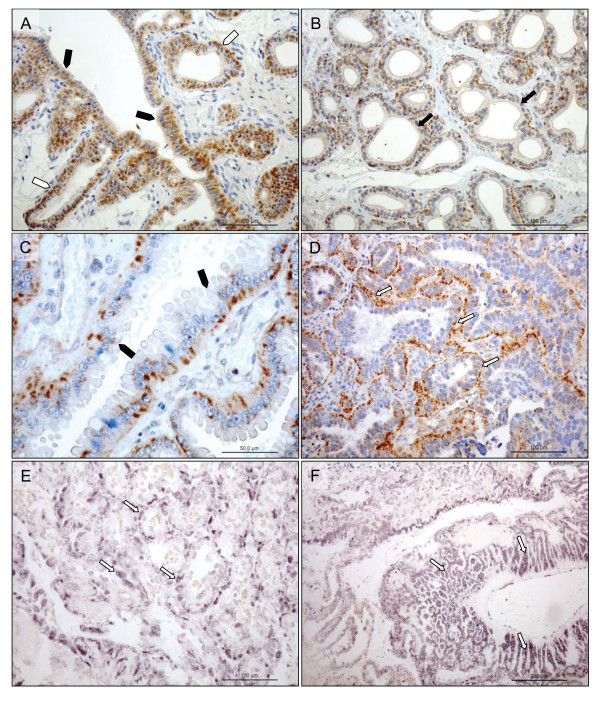
**Immunohistochemical (IHC) localization of PRLr in the uterus pre-implantation and in the utero/placental compartment**. (A, B) IHC localization of PRLr in canine endometrium at the pre-implantation stage of pregnancy. (C, D) IHC localization of PRLr within the utero/placental compartment (mid-gestation). (E, F) Localization of PRLr-mRNA in utero/placental compartment by in situ hybridization (ISH). (A, B) Pre-implantation PRLr is localized to the endometrial surface epithelial cells (solid arrowheads) and glandular epithelial cells of the superficial (open arrowheads) and deep (solid arrows) uterine glands. (C, D) Within the utero/placental compartment signals are localized to the endometrial glands including the superficial glands (solid arrowheads) (the so-called glandular chambers) and to the fetal trophoblast cells (open arrows). (E, F) The same localization pattern was observed at the mRNA-level by ISH. Thus, strong signals are localized to the trophoblast cells (open arrows) including those strongly invading maternal blood vessels at the base of placental labyrinth (open arrows).

Prior to implantation strong immunohistochemical signals were localized to surface and glandular epithelial cells (Figure 5A, B). Following the implantation and placentation the evenly distributed signals were targeted to the fetal trophoblast cells and to the epithelial cells of the glandular chambers (Figure 5C, D). A similar localization pattern was observed at the mRNA-level by in situ hybridization (Figure 5E, F).

### The effect of antiprogestin treatment on luteal and placental PRLr expression

**Figure 6:**
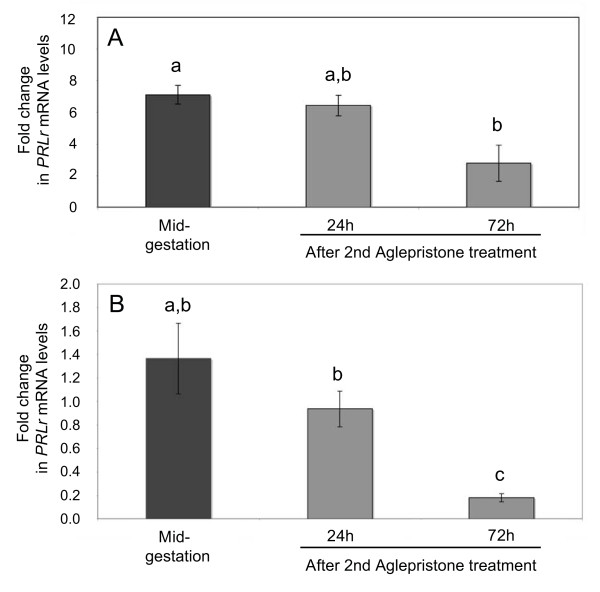
**The effects of antiprogestin treatment on luteal and placental expression of PRLr as determined by Real Time (TaqMan) PCR**. The fold changes in luteal (A) and utero/placental (B) PRLr mRNA expression compared with the mid-gestation group (non-treated control) are shown (mean ± SD). Bars with different asterisks in A differ at p < 0.01. In B "a, b" vs. "c" differ at p < 0.01 and "b" vs. "c" differ at p < 0.05.

Real Time PCR has been applied to test the effects of antiprogestin treatment on luteal and placental PRLr expression. Samples from the mid-gestation group served as non-treated control in the statistical evaluation with Dunnett's multiple comparison test. The luteal and utero/placental expression of PRLr was significantly (p < 0.01) decreased (2.5- and 7.6-fold, respectively) in response to the treatment with Aglepristone^® ^with more pronounced effects (p < 0.01) observed at 72 h than at 24 h after the 2nd. application (Figure 6A, B). The uterine PRLr expression was not affected by the antigestagen-treatment (p > 0.05) (Figure 4B).

## Discussion

As the canine placenta lacks steroidogenic activity, the establishment and maintenance of pregnancy depends on the provision of progesterone of luteal origin. Following the period of an apparent gonadotropic independence, especially from the second half of dioestrus, gonadotropic support is required for proper CL function. While PRL is the major factor in this process [10,11,20], LH seems to play a more indirect role, possibly as a mediator of PRL secretion [23]. Contrasting with this dominant role of PRL as a luteotropic factor within canine CL is the fact that the luteal regression and/or luteolysis take place in presence of increased LH and PRL concentrations in peripheral blood [6,14-16]. This remains also one of the most interesting peculiarities in mechanisms regulating canine reproduction.

There are several isoforms of PRLr known in different species, most of them representing alternative splice variants within the 3'ends of their cDNA sequences [26,27,32]. Here, using homology cloning, for the first time, a partial sequence of canine PRLr sequence displaying a high similarity (82 - 92%) with respective regions of other species (mouse, rabbit, pig, cattle, human) has been cloned and sequenced. It corresponds to the portions of the extra- and transmembrane domains conserved within the PRLr isoforms of other species, e.g exons 6-9 in mouse [27]. Further studies aiming to delineate complete cDNA of dog-specific PRLr and its potential isoforms should be considered. Nevertheless, the data presented provide the first molecular insight in the expression and localization of PRLr in CL from non-pregnant and pregnant dogs as well as in the utero/placental compartment. As determined on the mRNA- and protein-level, the luteal and utero/placental expression of PRLr was strongly time-related. In non-pregnant animals the luteal expression was highest at the beginning of dioestrus (day 15 p.o.); the decrease towards day 65 p.o. was significant (p < 0.05). This observation is seemingly in conflict with findings of Fernandes et al. [15], who reported about changes in concentration of unoccupied receptors for prolactin in canine luteal homogenates, which even though generally decreased after day 40 of luteal life-span, were statistically not significant. However, we anticipate that this contradiction may be due to differences in the detection limits of the methods used in the two studies. While in the past the determination of PRLr content in canine luteal tissues based on the binding of the radiolabeled purified human growth hormone to luteal homogenates [15], in the present study the very sensitive Real Time PCR was applied. The identity of selected amplicons with the hypophyseal PRLr was confirmed by sequencing.

Furthermore, the cycle-stage related expression of PRLr in CL of dioestric dogs resembled the course of P4 concentrations in peripheral plasma and may, hence, indicate that the provision of P4 in later stages of dioestrus could be controlled at the level of PRLr expression and/or function. Similarly, in pregnant animals the prepartal drop of PRLr-mRNA strongly resembled changes in luteal StAR and 3βHSD expression observed at the time of prepartal luteolysis [9]. Whether the decreased PRLr levels are the trigger or the result of luteolysis remains to be elucidated. However, since PRL acting through its receptor operates upstream of the steroidogenic machinery, the PRLr-mediated decline in luteal responsivity to PRL could be one of the triggers of luteolysis.

By investigating mechanisms involved in controlling reproductive function, recently, a new focus has been put on canine placenta as an important endocrine organ in this species [8,38]. Especially with respect to the prepartal luteolysis the placenta seems to be capable of actively responding to changes in the circulating P4 by activating of the enzymatic and hormonal signalling cascade leading to the prepartal output of luteolytic PGF2α. The knowledge, however, about mechanisms governing the development of canine placenta is very limited. Locally, at least in part, the process of decidualization and placentation in the bitch seems to be regulated by the PGTS2-derived arachidonic acid metabolites and their receptors [8,38].

One of the well characterized markers of the decidualization process is the expression of PRL and its receptor within the endometrium [40]. In the presented study the uterine expression of PRLr was localized to the surface and glandular epithelial cells. Thus, it can be speculated that in the dog PRLr might impact endometrial glandular secretory function utilizing endo- and/or paracrine mechanisms possibly playing a role in production of uterine milk (histiotrophe) as it has been suggested for human [41].

Furthermore, we conclude that the increase of PRLr-mRNA expression in the utero/placental compartment observed following the implantation must result from the upregulated PRLr levels within the placental compartment as the uterine expression of PRLr did not vary throughout the course of pregnancy. Immunohistochemistry and in situ hybridization localized expression of placental PRLr to epithelial trophoblast cells. Thus a role of PRL in the placental development possibly through influencing the invasion of fetal trophoblast cells, is strongly implied.

Recently the antigestagen-mediated effects on the luteal and placental prostaglandin-system have been shown [8,9]. Blocking of luteal and placental progesterone receptors during mid-pregnancy resulted in activation of the luteolytic cascade in a similar manner as observed during physiological prepartal luteolysis [8,9,42]. In this context, an interesting observation from this study is the effect of application of Aglepristone^® ^on PRLr expression in CL and utero/placental compartment, which mirrored the situation observed during prepartal luteolysis. Even if any functional interpretation at this moment would be premature, we found it an interesting observation suggesting a functional interrelationship between these two factors also with respect to the prepartal luteolysis.

## Conclusions

In summary, our study is the first to show the expression of PRLr in canine reproductive tissues. It puts a new focus on PRL not only as a key luteotropic factor involved in maintaining luteal steroidogenic activity in the dog but also as a factor potentially involved in regulating the uterine and placental function and hence having influence on embryo survival in this species.

A new insight into PRL-mediated regulatory mechanisms has been provided by showing the time dependent expression of PRLr in canine CL. This led us to speculate on the role of the PRLr-dependent cellular signalling pathways in regulating the provision of luteal P4 in the bitch.

## Competing interests

The authors declare that they have no competing interests.

## Authors' contributions

MPK: Idea of the study, collecting tissues samples, homology cloning procedure, statistical evaluation, interpretation of the data, manuscript writing. EM: Participated in study design, critical discussion of the data, editing of the manuscript. AG: RNA isolation, Real Time PCR experiments and immunohistochemical procedure. AB and FG: knowledge transfer, critical discussion of the data, editing of the manuscript. BH and SA: coordinating of animals experiments and collecting of tissue samples, knowledge transfer, critical discussion of the data, editing of the manuscript. IR: participated in study design, critical discussion of the data, knowledge transfer, editing of the manuscript. All authors read and approved the final manuscript.
